# Relationship Between the Japanese Physical Fitness Test and the 3-Minute Burpee Test

**DOI:** 10.7759/cureus.50710

**Published:** 2023-12-18

**Authors:** Yohei Yamashita

**Affiliations:** 1 Sports Medicine, Momoyama Gakuin University, Izumi, JPN

**Keywords:** 3-minute burpee test, endurance, muscle power, vo2max, new physical fitness test

## Abstract

Background

Japan has conducted a national physical fitness survey every year since 1964, when the first Tokyo Summer Olympics were held.

The survey is a test that evaluates eight physical fitness components: speed, total body endurance, instantaneous force, dexterity, muscle strength, muscular endurance, flexibility, and agility, but it requires specialized equipment and space, and it takes time to measure them all.

Aims

The purpose of this study was to examine the relationship between the 3-minute burpee test (3MBT), which has been reported to be associated with various physical fitness components, and a “new physical fitness test.”

Materials and methods

The relationship between the “new physical fitness test” and the 3MBT was examined in 122 college students (male=70, female=52) with no health problems.

Results

The “new physical fitness test” and the 3MBT showed a moderate relationship between the 20-m shuttle run (r = 0.685, p < 0.05), side stepping (r = 0.566, p < 0.05), standing long jump (r = 0.545, p < 0.05), grip strength (r = 0.461, p < 0.05), sit and reach (r = 0.305, p < 0.05), and sit-ups (r = 0.572, p < 0.05), indicating a moderate relationship.

Because this study used the Pearson product-rate correlation analysis, it is not possible to definitively assert a relationship with physical fitness factors.

Conclusions

However, the 3MBT is a very useful test because it requires whole-body strength, power, endurance, and aerobic capacity.

## Introduction

Japan has conducted a national physical fitness survey every year since 1964, when the first Tokyo Summer Olympics were held. Although there have been several changes in sampling strategies and physical fitness test batteries during this period, data from annual national physical fitness tests have been accumulated in Japan for over 50 years. While annual physical fitness tests are common for Japanese youth, this is not the case internationally, To the best of our knowledge, no other country conducts an annual national physical fitness survey as continuously as Japan does [1].

However, since only a few studies that used this physical fitness survey data set (Noi et al. (2002) [2], Nishijima et al. (2003a) [3], (2003b) [4], Kidokoro et al. (2020) [5], (2021) [6], (2023) [7], Tomkinson et al. (2020) [8], (2021) [9]) have been published in international peer-reviewed scientific journals, the Japanese system is largely unknown abroad. Currently, the “new physical fitness test” for 12-19-year-olds includes grip strength, sit-ups, sit and reach, sidestep, 20-m shuttle run (or endurance run), 50-m sprint, standing long jump, and handball throw.

In Japan, the Physical Fitness and Exercise Ability Survey has been conducted to clarify the current status of the nation's physical fitness and exercise capacity and has been widely used as basic data for the guidance and management of physical education and sports activities.

The “new physical fitness test” introduced in the 1999 Physical Fitness and Exercise Ability Survey has been fully revised in line with the current situation, taking into account changes in the physique of the population, advances in sports medicine and science, and the aging of the population, etc. It is expected that the “new physical fitness test” will be better understood and more meaningfully utilized so that people in the twenty-first century can lead healthy and vibrant social lives both physically and mentally. It is hoped that the “new physical fitness test” will be better understood and utilized in a meaningful way so that people living in the 21st century can lead healthy and vibrant social lives, both physically and mentally.

The purpose of each test is to assess eight physical fitness components: speed, total body endurance, instantaneous force, dexterity, muscle strength, muscular endurance, flexibility, and agility. Although the “new physical fitness test” is capable of assessing various physical fitness elements, it requires specific equipment and space, and it is time-consuming to measure all the tests.

Burpees were invented in 1930. Burpees are popular worldwide and are used in various online physical challenges [10-12]. Burpees are multi-joint exercises that use major muscle groups of the body, such as the pectoralis major, deltoids, quads, hamstrings, and trunk, and can be performed anywhere without a specific gym or equipment, in addition to having stimulation of metabolic mechanisms such as heart rate (HR) and blood lactate levels (BLa) [13-17]. There are also a variety of tests using the burpee, including 10 seconds [18], 20 seconds [19], 30 seconds [19], 60 seconds [19], and 3 minutes [19-24].

It has been reported that the short duration of about 10-20 seconds is mainly related to instantaneous force while 30-60 seconds is related to lean body mass and the number of cycles of burpees performed [18-24].

In particular, a 3-minute duration has been reported to be highly related to a variety of field tests, including maximal oxygen consumption (VO2max), countermovement jump height, isometric mid-thigh pull, isometric bench press, and lean body mass in women [19-24]. Therefore, it is expected that the relationship between the various field tests of the new fitness test will also be high.

Therefore, the purpose of this study was to determine the relationship between the “new physical fitness test” and the 3-minute burpee test (3MBT).

If there is a relationship between the 3MBT and the “new physical fitness test”, it is likely to be widely recognized as a test that can simultaneously train various physical fitness components without requiring specific equipment, location, or time.

## Materials and methods

The purpose of each test in the new physical fitness test (for males and females aged 12-19 years) is to assess eight physical fitness components: speed, total body endurance, instantaneous force, fine motor skills, muscle strength, muscle endurance, flexibility, and agility (Table 1).

**Table 1:** New physical fitness test (6-19 years)

Test Items	Physical strength evaluation factor	Kinetic property	
Grip strength	Muscular strength	Strength	
Sit-ups	Muscular strength, muscular endurance	Strength	Tenacity
Sit and reach	Flexibility	Softness of body	
Sidestep	Agility	Quickness	Timing
20 m shuttle run (or endurance run)	Total body endurance	Tenacity	
50 m sprint	Speed	Strength	Quickness
Standing long jump	Instantaneous force	Strength	Timing
Handball throw	Instantaneous force, dexterity	Strength	Timing

Each test was conducted according to the instructions of the Japan Sports Agency.

Grip strength

Grip strength was measured using a digital grip strength meter (GRIP D, TAKEI; Takei Scientific Instruments Co., Ltd., Tokyo, Japan). Hold the grip strength meter with the indicator facing outward and adjust the grip width by turning the knob so that the second joint of the index finger is at a right angle.

Standing upright, arms lowered naturally, gripping the grip as hard as they could, they alternated between right and left twice. The right and left were done in that order, and the same subject never did it twice in a row. Recordings were made in kilograms, rounded down to the nearest kilogram, and averaged over the left and right records, rounding down to the nearest kilogram.

Sit-ups

The subject lies supine on a mat, hands lightly folded in front of the chest, both knees at a 90° angle, and an assistant holds and secures the subject's knees.

On cue, the subject raised his upper body from the supine position until both elbows touched the thighs, and then immediately returned to the supine position for 30 seconds.

The number of times the upper body was raised (both elbows and thighs in contact) for 30 seconds was recorded, but not counted if the subject's back was not touching the mat when he returned to the supine position. Only one exercise was performed.

Sit and reach

Sit and reach were performed using a digital long-body forward bending measuring instrument (TAKEI). The initial posture was a long seated posture with both legs between the digital long seated forward bending measuring device and the back and buttocks firmly against the wall. The subject placed both hands shoulder-width apart, palms down, so that the middle of the palms rested on the front edge of the cardboard, and pulled the box fully forward with both hands, keeping the chest up, both elbows extended, and the back straight. The subject slowly bent forward and slid forward as far as possible without removing both hands from the digital long-bend extensometer. Care was taken not to bend the knees at this time, and the hands were removed from the digital longitudinal forward bending meter at the point of maximum forward bending. Measurements were taken twice, and the better of the two was adopted as the record, with measurements less than 1 cm rounded down.

Sidestep

A center line was drawn on the floor and two parallel lines were drawn 100 cm to the left and right of the center line. Participants stood across the center line, and on the "start" signal, sidestepped until they crossed or stepped on the right line (no jumping), returned to the center line, and sidestepped again until they crossed or touched the left line.

This exercise was repeated for 20 seconds and one point was awarded for each line crossed. The test was performed twice, and the better score was taken as the record.

20-m shuttle run

Two parallel lines were drawn 20 m apart, the participants stood on one of the lines, and the test was started by an electronic tone after a 5-second countdown that signaled the start of the test. One electronic tone was sounded at regular intervals, and by the time the next electronic tone sounded, the 20-m line was reached and the subject turned around on the spot when his/her foot crossed or touched the line. This action was repeated. If the line was reached before the electronic tone, the subject changed direction, waited for the electronic tone, and started running after the electronic tone sounded. The interval of the electronic sound set by the CD was slow at the beginning, but the interval of the electronic sound became shorter about every minute. In other words, the running speed increases approximately every minute, so the runner should try to keep up with the electronic sound intervals as much as possible. The test was terminated when the subject stopped running because he could no longer maintain the speed set by the CD or when he could no longer touch the line with either foot for two consecutive runs. In the case of a single delay from the electronic tone, the test could be continued as long as the subject was able to eliminate the delay in time for the next electronic tone. It should be noted that this test is a common field test in Japan, as it has been widely reported to be able to estimate maximal oxygen uptake.

Standing long jump

Both feet are lightly opened so that the toes of both feet are in line with the front edge of the step-off line, and both feet step forward at the same time. The distance of the straight line connecting the position closest to the step-off line among the positions where the body touched the mat and the position of the center of both feet before stepping off (the front edge of the step-off line) was measured. Two recordings were made and the better of the two was used. Recordings were made to the nearest centimeter, and fractions of less than one centimeter were rounded down.

3MBT (3-minute burpee test)

3MBT was performed with particular attention to correct posture, rather than aiming for speed of repetition. If a subject failed to perform any one of the series of movements correctly, it was considered a failure and was not counted. In particular, a plank posture with high or low hips and not clapping the hands over the head were also considered failures.

In addition, subjects practiced the 3MBT five times a week, similar to the method used in previous studies, in order to increase the reliability of the 3MBT [15,24].

Prior to administering the test, subjects performed a 10-minute warm-up consisting of jogging, jumping jacks, mountain climbers, and stretching. These exercises were performed to increase muscle temperature in the pectoral, deltoid, quadriceps, hamstrings, and trunk muscles used in burpees. After five minutes of rest from the end of the warm-up, the 3MBT was begun.

Participants

The subjects were 122 university students (70 males and 52 females), aged 18-22 years, affiliated with medical schools. The study was explained to the 320 students who had taken the required classes, and the 122 who gave their consent participated in the study. Although the participants exercised regularly, none of them belonged to a sports club or exercised for more than 120 minutes per week. Most had done burpees in the past and understood the movements, but none had worked on them regularly.

Preparation on the day of the test

To ensure safe administration of the new physical fitness test and the 3MBT, a physical condition check was conducted on the day of the test. The check items included fever (fever, chills, color), diarrhea (defecation, dehydration), body aches (joint pain, muscle pain, headache, chest pain, abdominal pain), lethargy (tiredness, fatigue), motivation (low motivation, worry), whether they slept well yesterday (poor sleep), and whether they ate yesterday's dinner and today's breakfast as usual (poor appetite). Participants also performed sufficient preparatory exercises just before the program implementation and were checked again for any abnormalities in their physical condition.

If any of the following symptoms were observed during the examination: feeling sick (poor mood), looking pale (poor complexion), breathing fast (respiratory status), pulsing fast (pulse rate), or moving slowly (movement status), the physical condition was checked to determine if the examination could be performed.

Statistical analysis

Performance in the 3MBT was correlated to performances in the various fitness tests using Pearson’s product-moment correlation coefficient (r). The interpretations of the strength of correlations were: small (0.1 |r| 0.29), moderate (0.3 |r| 0.49), large (0.5 |r| 0.69), very large(0.7 |r| 0.89), near-perfect (0.9 |r| 0.99) and perfect (|r| = 1.0) [25]. IBM SPSS Statistics 28 (IBM Corp., Armonk, NY, USA) was used for all statistical analyses, and the significance level of the tests was less than 5 % (two-tailed).

## Results

A total of 122 college students (male = 70, female = 52) with no health problems, average height 165.4±8.8 cm, average weight 56.7±10.1 kg, average skeletal muscle mass 41.9±8.5 kg, and average body fat percentage 22.8±7.3% were included (Table 2). The mean 3MBT was 61.9±15.8 cycles (male = 66.2±17.0 cycles, female = 55.7±11.4 cycles) (Table 2).

**Table 2:** Height, body mass, muscle mass, and body fat in the studied population 3MBT: 3-minute burpee test

Gender	Body height (cm)	Body mass (kg)	Muscle mass (kg)	Body fat (%)	No. of cycles (3MBT)
Subject average(n=122)	165.4±8.8	56.7±10.1	41.9±8.5	22.8±7.3	61.9±15.8
Male (n=70)	171.2±5.7	62.0±8.8	47.7±6.2	18.5±5.7	66.2±17.0
Female (n=52)	157.5±5.6	49.4±6.5	34.1±3.1	28.5±4.7	55.7±11.4

The results of the new fitness test were: 20-m shuttle run (male = 80.5±27.27, female = 47.1±13.56), VO2max (male = 42.8±7.53, female = 36.2±3.62), sidestep (male = 57±7.33, female = 50.5±6.1), standing long jump (male = 220.8±29.23, female = 168.7±18.15), grip strength (male = 37.6±10.31, female = 24.7±4.11), sit and reach (male = 50±10.77, female = 48.5±8.12), sit-ups (male = 29.9±6.36, female = 24.7±4.36) (Table 3).

**Table 3:** New physical fitness test averages by gender VO2max: maximal oxygen consumption

Gender	20-m shuttle run (rounds)	VO2max (ml/kg/min)	Sidestep (rounds)	Standing long jump (cm)	Grip strength (kg)	Sit and reach (cm)	Sit-ups (rounds)
Subject average (n=122)	66±27.8	40±7	54.1±7.5	198.3±36	32.1±10.4	49.4±9.7	27.7±6.2
Male (n=70)	80.5±27.3	42.8±7.5	57.0±7.3	220.8±29.2	37.6±10.3	50.0±10.8	29.9±6.4
Female (n=52)	47.1±13.6	36.2±3.6	50.5±6.1	168.7±18.2	24.7±4.1	48.5±8.1	24.7±4.4
Average for Japanese males aged 20-24	72.9±25.6 (n=1000)	42.4±11.5 (n=1000)	55.6±7.6 (n=1245)	227.3±23.5 (n=1254)	45.8±7.3 (n=1259)	45.3±10.4 (n=1273)	29.4±5.7 (n=1262)
Average for Japanese females aged 20-24	38.3±15.5 (n=798)	34.6±7.2 (n=798)	46.5±5.96 (n=987)	167.8±22.2 (n=1002)	27.8±4.9 (n=1012)	45.2±9.4 (n=1014)	21.3±5.6 (n=1012)

Relationships between the 3MBT and the new fitness test were found in men for the 20-m shuttle run (r = 0.660, large, p < 0.05), VO2max (r = 0.484, p = 0.05), side step (r = 0.539, large, p < 0.05), standing long jump (r = 0.555, large, p < 0.05), grip strength (r = 0. 404, p < 0.05), sit and reach (r = 0.395, p < 0.05), and sit-ups (r = 0.576, p < 0.05). Females were 20-m shuttle run (r = 0.579 p < 0.05), VO2max (r = 0.534, p = 0.05), side steps (r = 0.425, p < 0.05), and sit-ups (r = 0.317, p < 0.05). Relationships with all subjects and gender were represented (Table 4).

**Table 4:** Relationship between the 3MBT and the new physical fitness test Performance in the 3MBT was correlated to performances in the various fitness tests using Pearson’s product-moment correlation coefficient (r). 3MBT: 3-minute burpee test; VO2max: maximal oxygen consumption

Gender	20-m shuttle run	VO2max	Sidestep	Standing long jump	Grip strength	Sit and reach	Sit-ups
Subject average (n=122)	.685*	.558*	.566*	.545*	.461*	.305*	.572*
Male (n=70)	.660*	.484*	.539*	.555*	.404*	.395*	.576*
Female (n=52)	.579*	.534*	.425*	n.s.	n.s.	n.s.	.317*
							*=p<0.05

## Discussion

The purpose of this study is to determine the correlation between the 3MBT and the "new fitness test." The results showed that sit-ups (r = 0.572, p < 0.05), side step (r = 0.566, p < 0.05), 20-m shuttle run (r = 0.685, p < 0.05), standing long jump (r = 0.545, p < 0.05), grip strength (r = 0.461, p < 0.05), and sit and reach (r = 0.305, p<0.05) showed significant and moderate relationships [25].

The results of the new physical fitness test in our study were similar to those of Japanese subjects aged 20-24 years, indicating that the subjects in our study reflected the average fit Japanese person within the age group (Table 3). Thus, one would expect the same results as in the present study for those who do not engage in special training or exercise. Not only in our study but also in other reports, aerobic capacity has shown a significant relationship with VO2max [23-24].

Future work requires factor analysis to determine if the 3MBT is a tool to simultaneously assess whole-body strength, power, endurance, and aerobic capacity.

When compared to the 3MBT, a published international standard of performance, the number of times the subjects in this study performed the test (m = 66 and f = 55 times) was "average" and similar to that of non-exercising college students by Podstawski et al. [22].

Therefore, both the “new physical fitness test” and 3MBT performance in this study were similar to that of the average young adult, and the relationships obtained in our study are likely to be similar for many people.

By gender, women showed a relationship between 3MBT and 20-m shuttle run, VO2max, sidestep, and sit-ups. However, unlike men, no relationship was found for standing long jump, grip strength, and sit and reach. Compared to men, women's burpee movements may be more dependent on endurance. The average 3MBT (male = 66.2 ± 17.0, female = 55.7 ± 11.4), or one burpee every 2.7 seconds for men and one every 3.2 seconds for women. This is my guess, but men likely performed the 3MBT quickly based on their overall strength, including endurance and muscular strength. On the other hand, since women are considered to have weaker muscular strength than men, they may have performed it as an endurance exercise and could not do it quickly.

As a limitation of this study, we cannot clearly state the relationship with physical fitness factors due to the use of Pearson's product-rate correlation analysis, but it is clear that 3MBT is related to overall muscle strength, power, endurance, and aerobic capacity in men and women. In the future, we plan to conduct multiple regression analysis, factor analysis, and movement analysis to pursue the relationship and to clarify factors unrelated to muscle strength in women.

## Conclusions

The 3MBT was associated with whole-body strength, power, endurance, and aerobic capacity in men and aerobic capacity in women. Our results are likely to be similar for average young adults who are not engaged in any special physical fitness, strength building, or exercise. Although the differences between men and women and the usefulness of the 3MBT need to be clarified in the future, the 3MBT has the potential to be used as a tool to assess overall athletic performance, including muscle strength and aerobic capacity.
